# A novel probiotic therapeutic in a murine model of *Clostridioides difficile* colitis

**DOI:** 10.1080/19490976.2020.1814119

**Published:** 2020-09-20

**Authors:** Rita D. Shelby, Grace E. Janzow, Lauren Mashburn-Warren, Jeffrey Galley, Natalie Tengberg, Jason Navarro, Miriam Conces, Michael T. Bailey, Steven D. Goodman, Gail E. Besner

**Affiliations:** a Center for Perinatal Research, The Research Institute at Nationwide Children’s Hospital, Department of Pediatric Surgery, Nationwide Children’s Hospital, Columbus, OH, USA; b Center for Microbial Pathogenesis, The Research Institute at Nationwide Children’s Hospital, Columbus, OH, USA; c Department of Pathology, Nationwide Children’s Hospital, Columbus, OH, USA

**Keywords:** *C. difficile*, probiotics, lactobacillus reuteri, biofilm, therapeutic treatment, prophylactic treatment

## Abstract

*Clostridioides difficile* infection (CDI) is a common cause of antimicrobial-associated diarrhea. Probiotics have shown variable results in decreasing its incidence and severity. We examined the efficacy of *Lactobacillus reuteri* administered using a novel probiotic biofilm delivery system in the treatment and prevention of CDI in a murine model.

For prophylactic therapy, mice received an oral antibiotic cocktail followed by clindamycin injection, followed by probiotic administration (planktonic *vs*. biofilm state), followed by *C. difficile* oral gavage. For treatment therapy, mice received antibiotics and *C. difficile* first, followed by probiotic administration. Clinical sickness scores (CSS) and intestinal histologic injury scores (HIS) were assigned.

In the *Prophylactic Therapy* model, CSS: 67% of untreated mice exposed to *C. difficile* demonstrated CSS ≥ 6, which is consistent with *C. difficile* infection (*p*< .001 compared to unexposed mice). In mice treated with planktonic *Lr*, 55% had a CSS ≥ 6, but only 19% of mice treated with *Lr* in its biofilm state had CSS ≥ 6 (*p*< .001). Mice receiving *Lr* + DM-Maltose lost the least amount of weight compared to mice receiving saline (*p* = .004676) or to mice receiving *Lr* (*p*= .003185). HIS: 77% of untreated mice exposed to *C. difficile* had HIS scores ≥4, which is consistent with *C. difficile* infection. In mice treated with planktonic *Lr*, 62% had HIS ≥4, but only 19% of mice treated with *Lr* in its biofilm state had HIS ≥4. (*p*< .001). Additionally, mice treated with *Lr* in its biofilm state had better survival compared to untreated mice and to mice treated with planktonic *Lr* (*p* ≤ 0.05). Similar findings for weight loss, CSS, HIS and survival were obtained for *Treatment Therapy*.

A single dose of *Lactobacillus reuteri* in its biofilm state reduces the severity and incidence of experimental *C. difficile* infection when administered as both prophylactic and treatment therapy.

## Introduction

*Clostridioides difficile* (*C. difficile)* colitis is an antibiotic-associated infection that has led to a significant healthcare crisis in developed countries, affecting over 500,000 individuals annually and causing ~30,000 deaths each year worldwide.^1–3^ In the United States alone, *C. difficile* results in an economic burden of 4.8 USD billion in associated health-care costs annually.^1,4^ The typical pathogenesis of the disease is commonly understood to start when the host is first predisposed by gut microbiome dysbiosis. This is often a result of receiving single – or multiple-dose antibiotic treatment, but can result from other causes. Antibiotics lead to reduced microbial-produced secondary bile acids, and a resultant increase in primary bile acids. Some primary bile acids, such as taurocholate, increase germination of *C. difficile* spores,^5^ whereas secondary bile acids, such as ursodeoxycholic acid inhibits germination and vegetative *C. difficile* growth.^6^ Disease induction occurs via release of toxins A and B, and intestinal epithelial cell internalization of these toxins.^7,8^ The toxins cause inflammation and tissue infiltration necrosis in the mucous layer and colonic epithelial cells of the host. This damage manifests in the host as severe diarrhea.^7,8^ The most common treatments available to patients afflicted with *C. difficile* colitis involve antibiotic administration.^9^ However, despite antibiotic treatment, *C. difficile* colitis often recurs.^4,10^ An estimated 20–30% of adult patients experience recurrent *C. difficile* infection.^10^ This recurrence can be associated with increased morbidity, require additional medication or longer courses of therapy, and can result in increased patient mortality.^8,10^ In the most severe cases, care may also require fecal transplantation or colonic resection, but these procedures are not completely effective and are invasive.^8,10^

Given the potential for antibiotic treatment inefficacy,^4,10^ as well as the need for effective treatment options for recurrent *C. difficile* infection,^4,8,10^ further research is required. Probiotics have been previously shown to moderately restore eubiosis and reduce *C. difficile* infection in high-risk patients receiving antibiotics.^11^ However, clinicians have been hesitant to adopt probiotic therapies due to the lack of ease of administration and inconsistent study results regarding long-lasting protection against *C. difficile* colitis.^3^ Due to the increasing risk of *C. difficile* colitis disease and recurrence in recent years, a defined methodology to track the progression, diagnosis and treatment options for this disease is not only beneficial but imperative. Our previous work supported this notion by establishing novel clinical and histological scoring systems that allow for efficient and accurate identification of diseased animals in an experimental murine model of *C. difficile* colitis.^12^ We now incorporate these scoring systems to validate disease inoculation and to analyze the use of probiotics to prevent and treat *C. difficile* infection.

Previous studies from our laboratory have demonstrated that *Lactobacillus reuteri* (*Lr*) can be induced to form a biofilm by incubation with biocompatible dextranomer microspheres (DM).^13^ Furthermore, these microspheres can be loaded with beneficial cargo such as sucrose or maltose in order to increase biofilm production. Compared to *Lr* in its planktonic, free-living state, *Lr* in its biofilm state has significantly increased efficacy in protecting the intestines from neonatal necrotizing enterocolitis,^14,15^ another disease associated with intestinal dysbiosis. Based on these findings, we now examine the ability of *Lr* in its planktonic and biofilm state to protect the intestines from *C. difficile* colitis.

## Materials and methods

All experiments and procedures were reviewed and approved by the Nationwide Children’s Hospital Institutional Animal Care and Use Committee (IACUC Protocol # AR16-00095).

### Animals

A combination of mice (conventional C57BL/6 mice) bred in house and commercially obtained (Jackson Laboratories, Bar Harbor, ME) was utilized. Only fully weaned 8- to 10-week old mice were utilized. All mice were housed under identical conditions in groups of no less than 3 and no greater than 5 mice per cage. They were fed an irradiated, soy-free, low-fat rodent diet product # 2920x.10 (Harlan, Indianapolis, IN). UV-sterilized drinking water was provided *ad lib*. Animals were housed in a room designated only for *C. difficile* experimentation. All bedding and enrichment toys were autoclaved to avoid the introduction of outside microbes.

### Antibiotic administration

Antibiotics were administered over the course of 4 d in the form of a water cocktail as we have previously described.^12^ The water cocktail contained kanamycin (0.4 mg/mL), gentamicin (0.035 mg/mL), colistin (850 U/mL), metronidazole (0.215 mg/mL), and vancomycin (0.045 mg/mL), in the manner described by Julia et al.^16^ and by Chen et al.^17^ Antibiotics were purchased from Sigma-Aldrich (St. Louis, MO), reconstituted in sterile water, and provided to the mice *ad libitum* in their drinking water. The antibiotic concentrations were calculated based on an average weight of the mice used (20–25 gm), and expected water consumption over 4 d (4–6 mL/mouse/day). Twenty-four hours after antibiotic cocktail completion, an intraperitoneal (IP) injection of clindamycin (10 mg/kg) prepared in sterile water was administered.

### Clostridioides difficile

*Clostridioides difficile* was prepared as we have described previously.^12^ Vegetative (non-sporulated) *C. difficile* was prepared from a stock strain of VPI 10463 (ATCC 43255), which was purchased from the American Type Culture Collection (Manassas, VA). *C. difficile* was grown anaerobically in Modified Reinforced Clostridial Medium (ATCC medium 2107). To remove dissolved oxygen to facilitate *C. difficile* growth, the medium was degassed by briefly boiling while bubbling with N_2_ gas and reduced with 4 mM L-Cysteine, followed by pH adjustment to 6.8. *C. difficile* was grown in an anaerobic chamber in an atmosphere of 5% H_2_/10% CO_2_/85% N_2_ at 37°C for 48 hours. Bacteria were centrifuged for 5 minutes at 8000 x *g*, the media removed, and the pellet washed twice and resuspended with sterile degassed PBS in an anaerobic atmosphere. The final dosage per animal achieved was 1.5 × 10^7^ CFU of vegetative *C. difficile* in 150 μl. Individual aliquots were made under anaerobic conditions to minimize oxygen exposure during mouse treatment.

The optimum dosage of vegetative *C. difficile* needed to establish colitis was determined in preliminary experiments. This was accomplished by gastric gavage of varying colony forming units (CFUs) (10^6^, 10^7^, 10^8^ and 10^9^) of vegetative *C. difficile* after receipt of the oral antibiotic cocktail and IP clindamycin. In addition to varying CFUs, different incubation time periods were also tested, obtaining vegetative cultures at 24 hours, 36 hours, and 48 hours of growth. The dosage of 1.5 × 10^7^ CFU grown in culture medium for 48 hours prior to administration was chosen based on the findings that this dose led to *C. difficile* colitis in a substantial number of mice but was not uniformly lethal. Each mouse received 150 μL of *C. difficile* solution by gastric gavage.

### Lr biofilm preparation

Human-feces derived *L. reuteri* 23272 (American Type Culture Collection; ATCC, Manassas, VA) was grown overnight in de Man, Rogosa, and Sharpe (MRS) broth (Fisher Scientific, Pittsburgh, PA) at 37°C in 5% CO_2_. For planktonic *L. reuteri*, 1 × 10^9^ CFU/mL was pelleted and resuspended in sterile 0.9% saline prior to gastric gavage. For *L. reuteri* administered with unloaded microspheres, sterile dry dextranomer microspheres (Sephadex G-25 Superfine, GE Healthcare Bio-Sciences, Pittsburgh, PA) were hydrated in a sterile saline solution overnight. For *L. reuteri* administered with maltose-loaded microspheres, the microspheres were hydrated in a 1 M maltose solution in normal saline overnight. All microspheres were removed from the overnight solution via vacuum filter and aseptically transferred into a tube containing the resuspended bacteria. The bacteria were allowed to incubate with the microspheres for 1 hour at room temperature to facilitate binding. Each mouse received 200 μL of the bacterial solution by gastric gavage, for a final dose of 1 × 10^8^ CFU of *Lr*.

### Experimental model

**Figure 1.**
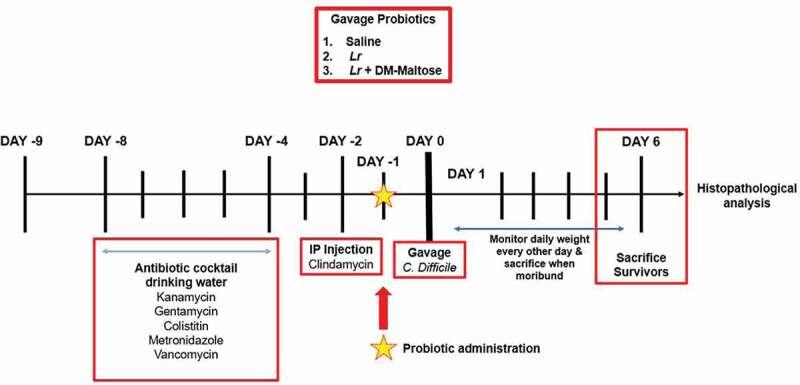
Experimental model for CDI prophylaxis. The experimental model spans 15 d. Following randomization into control or treatment groups, mice to be subjected to the *C. difficile* protocol were provided an antibiotic cocktail in sterilized drinking water over 4 d (days −8 to −4). Two days after oral antibiotic administration, mice received a single IP injection of clindamycin (day −2). 24 hours after IP clindamycin, mice randomized to treatment groups received one dose of: (1) saline (2) planktonic (*Lr*), or (3) *Lr+* DM-maltose. A single gastric gavage dose of *C. difficile* was administered 24 hours after prophylactic treatment. Control mice received no antibiotics, no probiotics, and no *C. difficile*. Mice were observed for 6 d post treatment.

**Figure 2.**
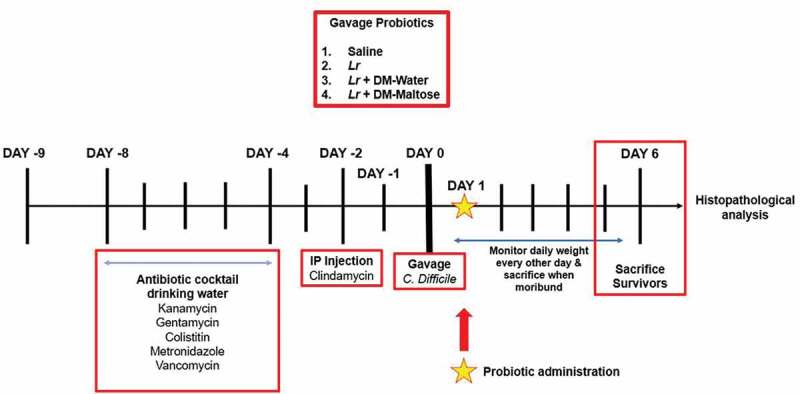
Experimental model for CDI therapy. The experimental model spans 15 d. Following randomization into vehicle control and treatment groups, mice to be subjected to the *C. difficile* protocol were provided an antibiotic cocktail in sterilized drinking water over 4 d (days −8 to −4). Two days after oral antibiotic administration, mice received a single IP injection of clindamycin (day −2). 24 hours after the IP clindamycin, mice received a single dose of *C. difficile*. 24 h later, mice randomized to treatment groups received one dose of: (1) saline (2) planktonic *Lr*, (3) *Lr* + DM-water, or (4) *Lr+* DM-maltose. Control mice received no antibiotics, no *C. difficile* and no probiotics. Mice were observed for 6 d post *C. difficile* inoculation.

The experimental scheme of the *C. difficile* colitis models for prophylactic and therapeutic treatments are illustrated in Figures 1 and 2 respectively. The experimental models span a 15-day time period. In the prophylaxis experiments, following randomization into control or treatment groups, mice to be subjected to the *C. difficile* protocol were provided an antibiotic cocktail in sterilized drinking water over 4 d (days −8 to −4). Two days after oral antibiotic administration, mice received a single IP injection of clindamycin (day −2). Twenty-four hours after IP clindamycin, mice randomized to treatment groups received one dose of (1) saline (2) planktonic (*Lr*), or (3) *Lr+* DM-maltose. A single gastric gavage dose of *C. difficile* was administered 24 hours after prophylactic treatment. Control mice received no antibiotics in their drinking water, saline gavage instead of probiotics gavage, and saline injection instead of *C. difficile* injection. Mice were observed for 6 d post treatment.

In the therapeutic experiments, following randomization into vehicle control and treatment groups, mice to be subjected to the *C. difficile* protocol again received an antibiotic cocktail over 4 d (days −8 to −4) followed 2 d later by a single IP injection of clindamycin (day −2). Twenty-four hours after the IP clindamycin, mice received a single dose of *C. difficile*. 24 h later, mice blindly randomized to treatment groups received one dose of (1) saline (2) planktonic *Lr*, (3) *Lr* + DM-water, or (4) *Lr+* DM-maltose. Control mice received no antibiotics in their drinking water, saline injection instead of *C. difficile* injection, and saline gavage instead of probiotics gavage. Mice were observed for 6 d post *C. difficile* inoculation. Mice were weighed every other day. Symptoms of disease (stool characteristics, weight loss, and decreased response to stimuli) were recorded and mortality was tracked. Animals judged to be in a moribund state were euthanized. Tissue samples from the cecum and colon were taken for histopathologic analysis.

### Changes in weight, clinical sickness scoring and histopathologic analysis

**Figure 3.**
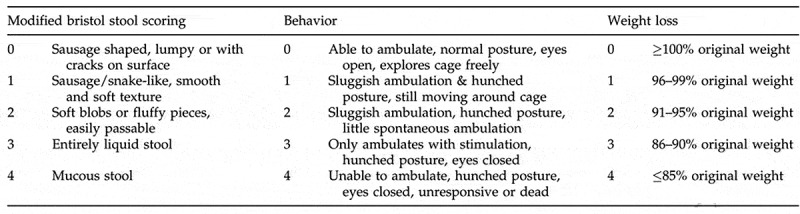
Clinical sickness scoring (CSS). Following *C. difficile* gavage, all mice were observed and assigned a daily clinical sickness score. Clinical sickness scoring is based on three categories: stool scoring, behavior and weight loss. Each category is scored in a range, from a score of 0 indicating no signs of sickness to a score of 4 indicating maximum signs of sickness. Scores assigned in each category are added into one cumulative CSS for each mouse. Combined final scores for each mouse range from 0 to 12, with the total score recorded in the final analysis. A CSS of 6 or greater is considered consistent with *C. difficile* colitis.^12^

**Figure 4.**
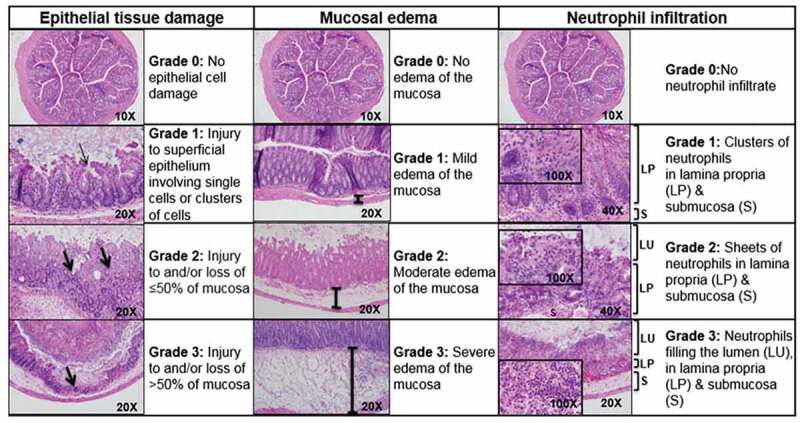
Histologic injury scoring (HIS). Mouse colon histology illustrating tissue damage in mice afflicted with *C. difficile* colitis. Scores in these categories range from no injury (0) to severe injury (3), and are combined into a cumulative HIS score ranging from 0 to 9. A HIS of ≥4 is indicative of *C. difficile* colitis.^12^ LU, lumen; LP, lamina propria, S, submucosa, thick arrow, level of mucosal injury; thin arrow, superficial epithelium injury.

Mice were weighed and assigned clinical sickness scores (CSS) and histologic injury scores (HIS) in a blinded fashion, as we previously described.^12^ CSS is based on clinical symptoms of stool characteristics, behavioral change, and percent weight loss (Figure 3). Each category is scored from 0 to 4, and the individual values are added to provide an overall score (Figure 3). A CSS of 6 or greater is considered consistent with *C. difficile* colitis, as we have previously reported.^12^ Animals achieving a CSS of ≥6 were euthanized. Upon sacrifice, the entire colon and cecum were collected for analysis. Histologic injury was graded based on epithelial tissue damage, amount of edema, and neutrophil infiltration (Figure 4). Each category was scored from 0 to 3 with the individual values added for an overall score. An HIS of ≥4 is indicative of *C. difficile* colitis, as we have previously reported.^12^

### Statistical methods

Differences in CSS and HIS were assessed using analysis of variance (ANOVA) with protected t-tests used as post hoc tests, using each individual score assigned to each individual animal. Survival was assessed by Kaplan–Meier survival analysis and tested using the log-rank test. All analyses were conducted using the SAS 9.4 statistical software program (SAS Institute, Cary, NC) with two-sided *p*-values <0.05 considered statistically significant.

## Results

### Lactobacillus reuteri administered prophylactically in its biofilm state decreases the incidence and severity of C. difficile colitis and improves survival

For these experiments, probiotics were administered as prophylaxis prior to *C. difficile* administration.

#### Weight loss

**Figure 5.**
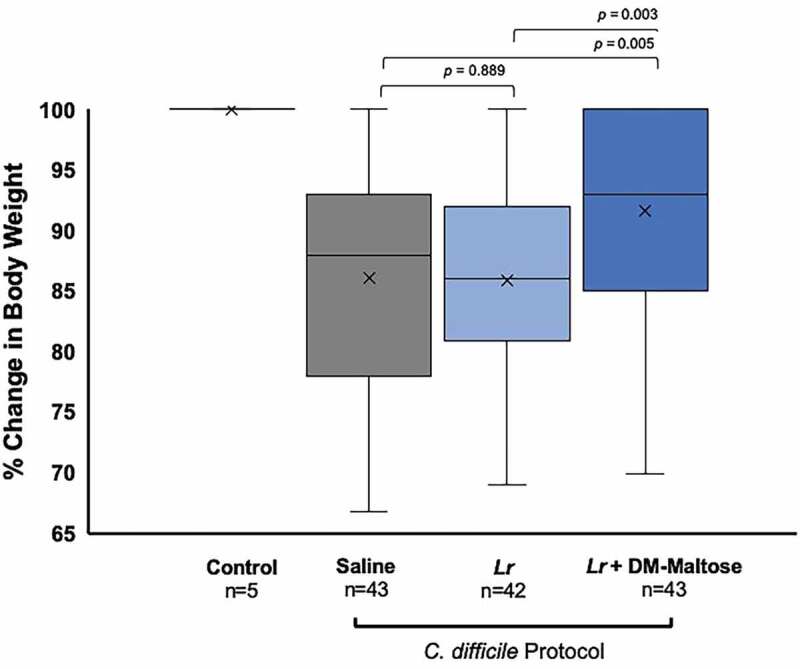
Percent of original body weight in CDI prophylaxis. Control mice received no antibiotics, no probiotics, and no *C. difficile*. Mice subjected to the *C. difficile* protocol were randomized to receive one dose of: (1) saline (2) planktonic *Lr*, or (3) *Lr +* DM-maltose. Weight was measured every other day. All 5 Control mice maintained their original body weights, indicated by the mean and bar range at 100%. Animals receiving *Lr* + DM-Maltose lost the least amount of weight compared to animals receiving saline (*p* = .004676) or compared to animals receiving *Lr* (*p*= .003185). There was no difference in percent of weight loss between animals receiving saline *vs. Lr* (*p*= .888959).

Control mice maintained their original body weight (Figure 5). Animals receiving *Lr* + DM-Maltose lost the least amount of weight compared to animals receiving saline (*p* = .005) and compared to animals receiving *Lr* (*p*= .003). There was no difference in the percent of weight loss between animals receiving saline *vs. Lr* (*p*= .889).

#### CSS

**Figure 6.**
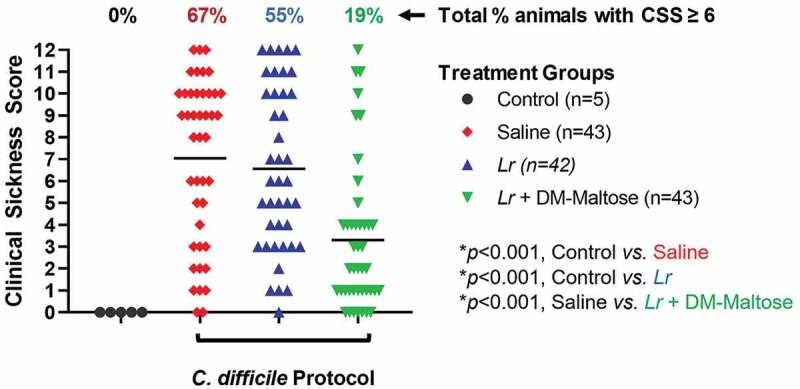
Clinical sickness score (CSS) grading with CDI prophylaxis. Control mice received no antibiotics, no probiotics, and no *C. difficile*. Mice subjected to the *C. difficile* protocol were randomized to receive one dose of: (1) saline (2) planktonic *Lr*, or (3) *Lr +* DM-maltose. Clinical sickness scores were assigned with daily observation. Total CSS for each animal ranges from 0 to 12 as illustrated in Figure 3. Each colored symbol represents one animal. Animals with scores ≥ 6 have CSS consistent with clinical *C. difficile* infection. The percentages at the top of each treatment group reflect the percent of animals with CSS ≥6 for each group. All *p* values are calculated based on individual animal scores.

Control mice that were not subjected to the *C. difficile* protocol did not develop signs of sickness. Compared to control mice, 67% of mice subjected to the *C. difficile* protocol that received saline only had CSS scores >6, consistent with *C. difficile* infection (*p* < .001) (Figure 6). Fifty-one percent of those animals had severe clinical signs of sickness with CSS ≥9. There was no significant difference in the incidence of clinical sickness in mice subjected to the *C. difficile* protocol that had prophylaxis with planktonic *Lr* compared to mice that received saline only (*p*= .553). However, compared to mice that received saline only, mice that received prophylaxis with a single dose of *Lr* + DM-maltose (*Lr* in its biofilm state) had a significantly lower incidence of CSS score ≥6 (*p*< .001).

#### HIS

**Figure 7.**
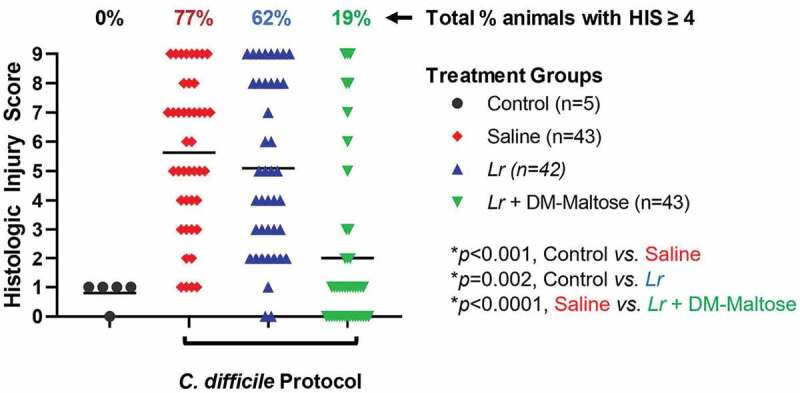
Histologic injury score (HIS) grading with CDI prophylaxis. Control mice received no antibiotics, no probiotics, and no *C. difficile*. Mice subjected to the *C. difficile* protocol were randomized to receive one dose of: (1) saline (2) planktonic *Lr*, or (3) *Lr +* DM-maltose. Total HIS for each animal ranges from 0 to 9 (illustrated in Figure 4). Each colored symbol represents one animal. Animals with scores ≥ 4 have HIS consistent with *C. difficile* infection. The percentages at the top of each treatment group reflect the percent of animals with HIS ≥ 4 for each group. All *p* values are calculated based on individual animal scores.

Control mice that were not subjected to the *C. difficile* protocol did not develop histologic injury. Compared to control mice, 77% of mice subjected to the experimental *C. difficile* protocol that received saline only had HIS scores ≥4, which is consistent with *C. difficile* infection (*p* < .001) (Figure 7). Forty-seven percent of those animals had a severe histological injury with HIS ≥7. There was no significant difference in the incidence of histologic injury in mice subjected to the *C. difficile* protocol that had prophylaxis with planktonic *Lr* compared to mice that received saline only (*p*= .379). However, compared to mice that received saline only, mice that received prophylaxis with a single dose of *Lr* + DM-maltose (*L*r in its biofilm state) had a significantly lower incidence of HIS ≥ 4 (*p* < .001).

#### Survival

**Figure 8.**
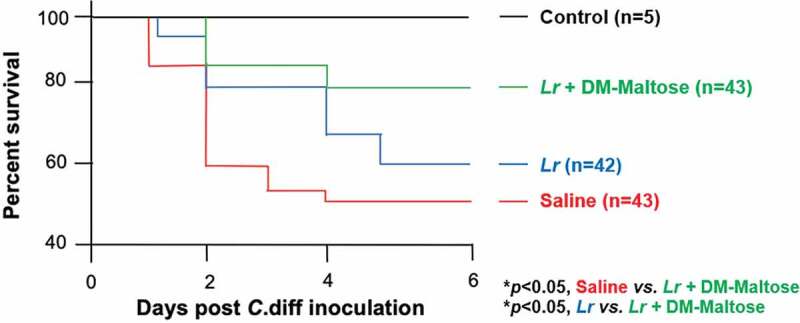
Survival in CDI prophylaxis. Control mice received no antibiotics, no *C. difficile* and no probiotics. Mice subjected to the *C. difficile* protocol were randomized to receive one dose of: (1) Saline (2) Planktonic *Lr*, (3) *Lr* + DM-Water or (4) *Lr +* DM-Maltose. Kaplan–Meier survival curve demonstrating percent death and survival of animals in each treatment group over the time course of the experiment.

Kaplan–Meier survival analysis revealed a significant difference in animal survival in mice exposed to the prophylactic *C. difficile* protocol treated with saline only compared to mice that were treated with a single dose of *Lr* + DM-Maltose at the end of the experiment on Day 6 (48% *vs*. 78%; *p*< .05) (Figure 8). There was no significant difference in the survival of mice treated with saline only compared to planktonic *Lr* treated mice within this experimental timeframe (48% *vs*. 59%, *p*> .05) (Figure 8).

### Lactobacillus reuteri administered therapeutically in its biofilm state decreases the incidence and severity of C. difficile colitis and improves survival

For these experiments, probiotics were administered as a treatment after *C. difficile* infection was already established.

#### Weight loss

**Figure 9.**
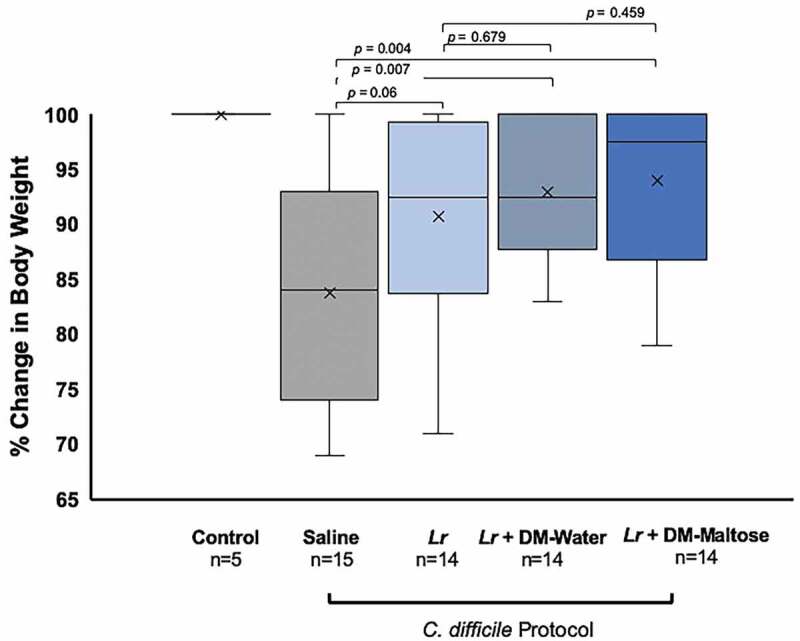
Percent of original body weight in CDI therapy. Control mice received no antibiotics, no probiotics, and no *C. difficile*. Mice subjected to the *C. difficile* protocol were randomized to receive one dose of: (1) saline (2) planktonic *Lr*, (3) *Lr +* DM-maltose, or (4) *Lr +* DM-maltose. Weight loss was measured every other day. All 5 Control mice maintained their original body weights, indicated by the mean and bar range at 100%. Animals receiving *Lr* + DM-Maltose and *Lr* + DM-Water lost the least amount of weight compared to animals receiving saline (*p*= .0043 and 0.0067, respectively). There was no difference in percent of weight loss between animals receiving saline *vs. Lr* (*p*= .0.0628). There was no difference in percent of weight loss between animals receiving *Lr* + DM-Maltose or *Lr* + DM-Water *vs. Lr* (*p*= .4591 and *p*= .6790, respectively).

Control mice maintained their original body weight. Animals receiving *Lr* + DM-Maltose and *Lr* + DM-Water lost the least amount of weight compared to animals receiving saline (*p*= .0043 and 0.0067, respectively), whereas there was no difference in percent weight loss between animals receiving *Lr vs*. saline (*p*= .0628) (Figure 9). There was no difference in the percent of weight loss between animals receiving *Lr* + DM-Maltose or *Lr* + DM-Water *vs. Lr* (*p*= .4591 and *p*= .6790, respectively).

#### CSS

**Figure 10.**
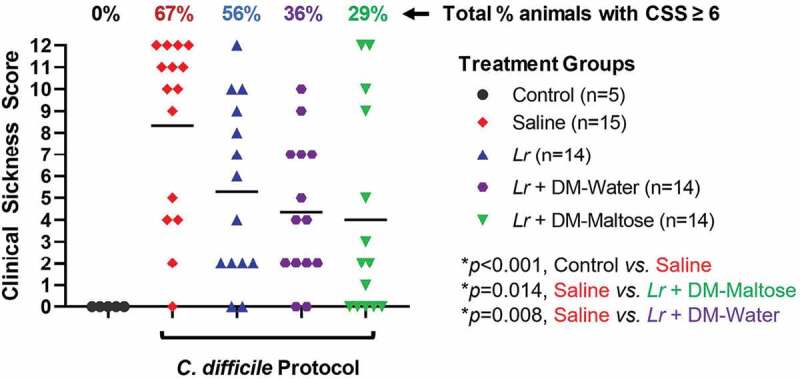
Clinical sickness score (CSS) grading with CDI therapy. Control mice received no antibiotics, no *C. difficile* and no probiotics. Mice subjected to the *C. difficile* protocol were randomized to receive one dose of: (1) saline (2) planktonic *Lr*, (3) *Lr* + DM-water or (4) *Lr +* DM-maltose. Clinical sickness scores were assigned with daily observation. Total CCS for each animal ranges from 0 to 12 as illustrated in Figure 3. Each colored symbol represents one animal. Animals with scores ≥ 6 have CSS consistent with clinical *C. difficile* infection. The percentages at the top of each treatment group reflect the percent of animals with CSS ≥6 for each group. All *p* values are calculated based on individual animal scores.

Control mice that were not subjected to the *C. difficile* protocol did not develop signs of sickness. Compared to control mice, 67% of mice subjected to the experimental *C. difficile* protocol that received saline only had CSS scores ≥6, consistent with *C. difficile* infection (*p* < .001) (Figure 10). All of these animals had CSS scores consistent with severe signs of sickness (CSS ≥9). There was no significant difference in the incidence of clinical sickness scores in mice subjected to the *C. difficile* protocol that were treated with planktonic *Lr* compared to mice that received saline only (*p*= .056). However, compared to mice that received saline only, mice that received a single dose of *Lr* + DM-water had a decreased incidence of CSS ≥ 6 (*p*= .008), and mice treated with *Lr* + DM-maltose had a further decrease in the incidence of CSS ≥ 6 (*p*= .014).

#### HIS

**Figure 11.**
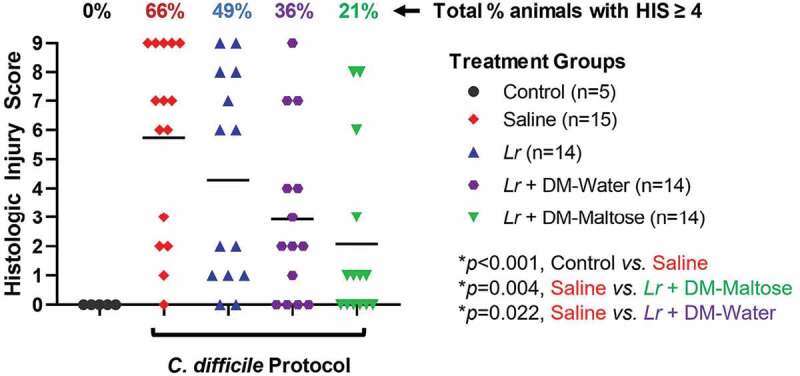
Histologic injury scores (HIS) grading with CDI therapy. Control mice received no antibiotics, no *C. difficile* and no probiotics. Mice subjected to the *C. difficile* protocol were randomized to receive one dose of: (1) saline (2) planktonic *Lr*, (3) *Lr* + DM-water or (4) *Lr +* DM-Maltose. Total HIS for each animal ranges from 0 to 9 (illustrated in Figure 4). Each colored symbol represents one animal. Animals with scores ≥ 4 have HIS consistent with *C. difficile* infection. The percentages at the top of each treatment group reflect the percent of animals with HIS ≥4 for each group. All *p* values are calculated based on individual animal scores.

Control mice that were not subjected to the *C. difficile* protocol did not develop histologic injury. Compared to control mice, 66% of mice subjected to the *C. difficile* protocol that received saline only had HIS scores ≥4, consistent with *C. difficile* infection (*p* = .002) (Figure 11). Fifty-three percent of those animals had HIS scores consistent with severe histological injury with HIS ≥7. There was no significant difference in the incidence of histologic injury in mice subjected to the *C. difficile* protocol that were treated with planktonic *Lr* compared to mice that received saline only (*p*= .263). However, compared to mice that received saline only, mice that received a single dose of *Lr* + DM-Water had a decreased incidence of histologic injury ≥4 consistent with *C. difficile* infection (*p*= .022), and mice treated with *Lr* + DM-maltose had a further and significant decrease in histologic injury ≥4 (*p = *.004).

#### Survival

**Figure 12.**
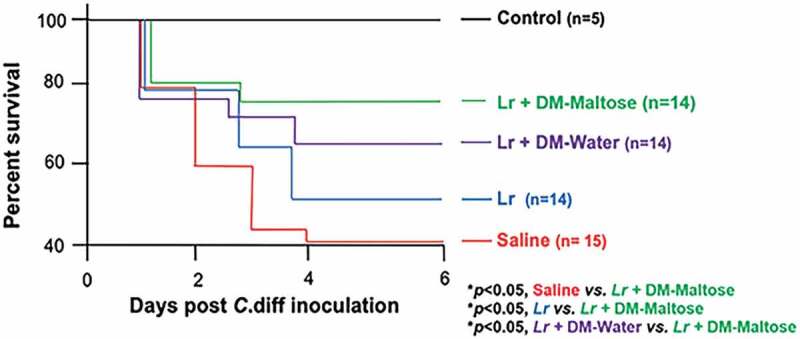
Survival in CDI treatment. Control mice received no antibiotics, no *C. difficile* and no probiotics. Mice subjected to the *C. difficile* protocol were randomized to receive one dose of: (1) Saline (2) Planktonic *Lr*, (3) *Lr* + DM-water or (4) *Lr +* DM-Maltose. Kaplan–Meier survival curve demonstrating percent death and survival of animals in each treatment group over the time course of the experiment.

Kaplan–Meier survival analysis revealed a significant difference in animal survival between saline-treated mice and mice that received a single dose of *Lr* + DM-Maltose therapeutically by the end of the experiment on Day 6 (42% *vs*. 78%; *p*< .05) (Figure 12). There was also a significant difference in survival between animals that received *Lr* + DM-maltose and those that received planktonic *Lr* at Day 6 (78% *vs*. 50%; *p*< .05). There was no significant difference in survival of mice that received a single dose of *Lr* + DM-maltose compared to those that received *Lr* + DM-Water by Day 6 (78% *vs*. 66%, *p > *.05).

## Discussion

The incidence of *C. difficile* colitis is rising in both pediatric and adult populations.^1,18^ Despite this, the management of *C. difficile* colitis still requires optimization. At present, the most common instigator that leads to the development of *C. difficile*, antibiotics, is also the first-line treatment option for both initial and recurrent *C. difficile* colitis.^8,11,18^ However, in spite of these established therapies, many patients often have a number of recurrent infections that are resistant to antibiotic therapy. Additionally, disease in some patients can rapidly escalate in severity, leaving no other treatment option but surgical intervention.^11,19,20^

With the continued rise in the incidence of initial and recurrent disease, as well as associated complications, there has been an increased interest in the development of alternative therapies. In particular, probiotics have been of significant interest in the treatment and prevention of *C. difficile* colitis.^3,11,21^ Probiotics are theoretically efficacious because of their potential to restore eubiosis to the gastrointestinal microbiota after disruption by antimicrobials.^22^ There have been several studies recently published supporting the use of probiotics for their protective effect against *C. difficile* infection.^3,23^ However, to date, only moderate efficacy of the several probiotics used has been obtained.^24–27^ It is important to note that in all of these studies and analyses, probiotics were evaluated in the *prevention* of *C. difficile* colitis and not in the treatment of the disease. Furthermore, in all of these studies, probiotics were administered in their planktonic state and required repeated doses to demonstrate efficacy.^28,29^ In the current study, we have demonstrated that the administration of one single dose of *Lr* in its biofilm state can significantly reduce the incidence and severity of *C. difficile* colitis when administered either prophylactically or therapeutically.

*Lr* is present in the healthy human intestinal tract and was originally isolated from human breast milk.^30,31^ The current studies utilized clade II *Lr* (ATCC 23272) originally isolated from human stool. This strain of *Lr*, among others, has antimicrobial activity conferred by its ability to convert glycerol into the antimicrobial compound reuterin, and it has anti-inflammatory properties due to its ability to produce histamine and to modulate cytokine production.^32,33^ *Lr* also readily forms a biofilm and has a significant affinity for the cross-linked dextran in DM. Because of these properties, as well as our own experience with this probiotic formulation in the prevention of necrotizing enterocolitis (NEC),^14,15^ we chose to use *Lr* for our experimental model.

In the current study, we found no efficacy of a single dose of *Lr* administered in its planktonic state either prophylactically or therapeutically. On the other hand, we were able to demonstrate efficacy with the administration of just one single dose of *Lr* administered in its biofilm state. Other strains of planktonic *Lr* have demonstrated various levels of efficacy with repeated multi-dose delivery in other studies.^3,24,34^ Britton et al. demonstrated that *Lr* as ‘precursor-directed antimicrobial therapy’ is effective in targeting CDI. In addition, they argue that antimicrobial resistance can be leveraged in the natural human microbiome response to evade *C. diff* – demonstrating the efficacy of administering *Lr* as a prophylactic probiotics.^23^ This is similar to our prophylactic results; however, in our studies, the best efficacy was noted in our novel formulation of *Lr* adhered to maltose-loaded DM, where there is the greatest biofilm formation. We have previously shown that *Lr* in its biofilm state demonstrates prolonged survival in acidic environments and improved adherence to intestinal epithelial cells *in vitro*^13,15^ and protects the intestines from NEC *in vivo*.^14,15^ In our NEC studies, augmentation of biofilm formation by adherence of *Lr* to DM loaded with sucrose or maltose leads to increased intestinal protection,^14^ and conversely, decreasing the ability of *Lr* to produce a biofilm by using a genetically altered strain of *Lr* (Δ*gtfW*) decreases intestinal protection.^14^

The adherence of *Lr* to DM to promote biofilm formation is a central component of its improved protective effect seen in this study. *Lr* relies on a novel extracellular glucosyltransferase (GtfW), rare among bacteria, that does not rely on sugar nucleotide intermediates but uses the energy of existing glycosidic linkages to generate chains of polysaccharides. Indeed, *Lr* adheres to DM via the glucan-binding domain of the GtfW enzyme^14^ as well as the ability to directly use maltose to make glucan homopolymers that facilitate binding to DM. Importantly, we have previously shown that *Escherichia coli, Salmonella typhimurium*, and *Clostridioides difficile* do not detectably bind to DM,^13^ allaying any concerns that the administration of DM might provide a platform for increased biofilm formation in pathogenic bacteria.

A limitation of the current work is that an analysis of the gut microbiome after antibiotic administration *C. difficile* inoculation or probiotic therapy was not performed. However, we have investigated the effect of *L. reuteri* in its biofilm state on the incidence and severity of necrotizing enterocolitis (NEC), and in those studies we were able to demonstrate a change in the microbiome when *L. reuteri* was administered in its biofilm compared to its planktonic state.^14^ Based on this, a change in microbiome may also play a role in our *C. difficile* studies, and will be addressed in future studies.

The novel formulation presented here represents a potential exciting development toward improving probiotic therapy for a devastating public health problem. Our results showing that *Lr* in its biofilm state is superior to planktonic *Lr* provide great insight into future potential therapeutics.

## Conclusion

*Lr* administered in its biofilm state exhibits a protective effect against *C. difficile* colitis when administered both prophylactically and as a treatment, with just one single dose. These results support the future translation of this treatment to the bedside given the dual efficacy of this probiotic.
